# Anxiety among urban, semi-urban and rural school adolescents in Dhaka, Bangladesh: Investigating prevalence and associated factors

**DOI:** 10.1371/journal.pone.0262716

**Published:** 2022-01-21

**Authors:** Afifa Anjum, Sahadat Hossain, M. Tasdik Hasan, Md. Elias Uddin, Md. Tajuddin Sikder

**Affiliations:** 1 Department of Public Health and Informatics, Jahangirnagar University, Savar, Dhaka, Bangladesh; 2 Health System and Population Studies Division (HSPSD), International Centre for Diarrhoeal Disease Research, Bangladesh (icddr,b), Dhaka, Bangladesh; 3 Department of Behavioural Science and Health, Institute of Epidemiology and Health Care, University College London (UCL), London, United Kingdom; 4 Department of Public Health, State University of Bangladesh, Dhaka, Bangladesh; 5 Jeeon Bangladesh Ltd., Dhaka, Bangladesh; 6 Department of Primary Care & Mental Health, University of Liverpool, Liverpool, United Kingdom; 7 Department of English, University of Dhaka, Dhaka, Bangladesh; Shahjalal University of Science and Technology, BANGLADESH

## Abstract

**Background:**

Anxiety disorder is one of the emerging public health problems in many low- and middle-income countries (LMICs). Likewise, in Bangladesh, a growing number of adolescents are experiencing such symptoms though we have very limited research evidence available. The purpose of this study was to investigate the prevalence of anxiety and the factors associated with this condition among urban, semi-urban, and rural school adolescents in Bangladesh.

**Methods:**

This cross-sectional study used a two-stage cluster sampling procedure. A self-administered questionnaire was conveyed to 2355 adolescents from nine secondary schools of Dhaka, Bangladesh. Of the respondents, 2313 completed the seven-item Generalized Anxiety Disorder (GAD-7). Besides, sociodemographic information, self-reported body image as well as modification of Leisure Time Exercise Questionnaire (LTEQ) and WHO Global PA Questionnaire (GPAQ) were used to determine the sociodemographic and lifestyle factors associated with anxiety among adolescents.

**Results:**

A total of 20.1% of adolescents were experiencing moderate to severe anxiety; of them, a significantly higher proportion (49.9%) of female adolescents were suffering more than males (40.1%). Furthermore, age, student’s grade, father’s educational level, number of family members, and residential setting were found to be significantly associated with anxiety among adolescents. In terms of lifestyle factors, irregular physical activity (AOR: 1.31; 95% CI: 1.05–1.63), high screen time (AOR: 1.51; 95% CI:1.21–1.88), sleep dissatisfaction (AOR: 3.79; 95% CI: 3.02–4.76), and underweight body image (AOR: 2.37; 95% CI:1.70–3.28) were found to be significantly associated with anxiety among school adolescents of urban, semi-urban, and rural residential settings.

**Conclusions:**

Anxiety is prevalent among urban, semi-urban, and rural school adolescents in Dhaka, Bangladesh. To lessen this prevalence of anxiety among Bangladeshi adolescents, evidence-based health programs- healthy school trials—and policies should therefore be taken based on the findings of this study.

## Introduction

Anxiety disorders are widespread and consist of debilitating symptoms that often begin in childhood, adolescence, and early adulthood [1]. They vary from developmentally normative or stress-induced acute and chronic anxiety and impair day-to-day functioning [2]. Many anxiety disorders affect mostly females compared to males, and in some cases, females are about twice at risk of developing them [3]. Anxiety disorders also co-occur with severe depression, alcohol and other drug use disorders, and personality disorders. When these are left untreated, they may occur recurrently [2].

According to the World Health Organization, anxiety disorders hold a great proportion of adolescent mental health diseases and it is considered to be the most common type of mental illnesses among adolescents [4]. Findings of studies on the global burden of diseases showed that about 5.5% female and 3% male adolescents aged 15–19 years suffer from anxiety disorders [5]. Anxiety disorders are prevalent among adolescents in the developed and developing countries in the world [6–8]. The study of Ghandour et al. reported that in the USA, the prevalence of anxiety problems among children and adolescents aged 3–17 years was 7.1% [9]. In an Iranian study, Mohammadi et al. found that the prevalence of social anxiety disorder among children of 6–18 years was about 2% [10]. Essau et al. found that the adolescents of England were found to be suffering significantly higher from anxiety symptoms than their Japanese counterparts [11]. In terms of the lower middle income countries (LMIC), a study conducted in Uganda found that the prevalence of anxiety disorders among children and adolescents aged 3–19 years was 26.6% [12], while in another study in Nigeria, it was found that the twelve-month prevalence of all anxiety disorders among adolescents aged 13–18 years was 15.0% [13]. Khalid et al. reported that among 1124 Pakistani adolescents belonging to the age-group of 11–18 years, the prevalence of anxiety was 21.4% [14]. Furthermore, a study conducted in Sri Lanka among 445 adolescents aged 14–18 years found that the prevalence of severe anxiety was 28% [15]. In addition, during the COVID-19 pandemic, an increase in the prevalence of adolescence anxiety has been observed around the world [16, 17].

Studies revealed that anxiety in early adolescence triggers homotypic anxiety in late adolescence. It is quite evident from different research findings that anxiety disorders are quite prevalent among children and adolescents along with different internalizing and externalizing disorders [18]. In the higher income countries, different factors are found to be associated with anxiety disorders among adolescents, for example, gender, order of birth, parental educational level, number of siblings and others [19, 20]. Besides, anxiety among adolescents differs along urbanicity and rurality. Different social, ecological, and familial factors play roles in this regard. Studies also showed that distance from the parents, family, near and dear ones, school problems, peer conflict and peer isolation, and stressful life conditions may contribute to anxiety, stress and depression as well as behavioral problems in adolescents [21]. In spite of being one of the deadliest ailments, it has not yet received as much attention as depression and mood and psychotic disorders. Therefore, it is responsible for reduced productivity, higher morbidity and mortality, and spurt in alcohol and drug abuse among a large number of people [18, 22].

Anxiety is quite prevalent among Bangladeshi youths. According to national mental health survey 2018–19, in Bangladesh the prevalence of anxiety disorders among children of 7–17 years was 4.5% [23]. A study which used DASS-21 for determining presence of anxiety among youths found that the alpha value of anxiety was 0.80 (95% CI: 0.76 ~ 0.84) [24]. In another study, it was reported that youths in Bangladesh who perceived their health condition as poor were more likely to be suffering from anxiety (AOR: 4.365; 95% CI: 2.599–7.332) [25]. Among 2,989 adolescents (data obtained from 2014 Bangladesh Global School-based Student Health Survey (GSHS), supported by the World Health Organization and the US Centers for Disease Control and Prevention) the prevalence of anxiety disorder was reported to be 4.7%, with a higher prevalence among females [8]. In a recent study, Saiful et al. found that among adolescents of 13–18 years the prevalence of moderate to severe anxiety was about 18% which is really a concerning fact for the coming days [26]. But the fact is none of the mentioned studies conducted in the Bangladesh context reported a comprehensive picture of the prevalence of anxiety among the adolescents of urban, semi-urban and rural areas. Thus, the data regarding mental health problems of rural and semi-urban areas remain unnoticed and the mental health services keep their focus on urban context by clustering the services in urban areas [27]. Therefore, this study was conducted on the school adolescents of urban, semi-urban and rural areas to find out the prevalence of anxiety and factors associated with this.

## Methods

### Study design and setting

This cross-sectional study was conducted between January 2019 and February 2020 in three different areas—urban, semi-urban and rural. In this study, Dhanmondi thana in Dhaka city was chosen as the urban area, Savar thana, 24 kilometres northwest of Dhaka city, was the semi-urban area, and Dhamrai upazila, which is about 40 kilometres north west of the capital Dhaka, was the rural area. Given the intra-thana (administrative division of a district*)* homogeneity of the population, these three areas were chosen for this study.

### Study procedures

Initially all the secondary schools of the urban, semi-urban and rural areas were listed, and it was found that the number of secondary schools in Dhanmondi, Savar and Dhamrai were 16, 14 and 16 respectively. Three schools were chosen randomly from each region by taking into account the diverse socio-economic context of the participants and their accessibility to the research team. The study population in this study consisted of adolescents of grades 8–10 (12–17 y), since grades 6–10 constitute the secondary education at schools in Bangladesh. Following a two-stage cluster sampling technique with a 10% non-response rate, a sample of 2355 was calculated for this study.


$$n=\frac{z^{2}pq}{d^{2}}\times 2$$

$$n=\frac{{\left(1.96\right)}^{2}\times 0.366\times (1-0.366)}{{\left(0.05\right)}^{2}}\times 2$$

n=713.14

n≈713

Here,

n = Total sample size

z = 1.96 (for 95% Confidence Interval)

p = 0.366 (previous mental disorder rate among school adolescents)

q = (1-p), = (1–0.366)

d = 0.05 (where 5% margin of error was accepted)

DE = Cluster design effect = 2

Prior to field data collection, the research team collected a student list of all the enrolled students of grades 8, 9 and 10 from the school authorities. After that, with the approval of the headmaster/principals of the schools and then the teachers of respective classes, the research team went to classrooms and explained the rationale and aim of the study to the students. Students were also informed that they were required to seek permission from their parents for taking part in the study. After the study team had obtained the assurance of individual assent and informed consent from the students and their parents, the survey questionnaires were distributed among the students. 2313 students completed the survey questionnaire (98% response rate) in the classroom under the direction of the lead researcher. A teacher and a member of the study team were both present in the classroom to track the progress of the survey and to answer any questions or to resolve any queries of the participants. Students who did not consent to take part did activities of their choice during the survey.

### Measures

#### Anxiety (outcome)

In order to identify probable cases of generalized anxiety disorder (GAD) and measure the level of severity of anxiety disorders of the participants, one most popularly used 7-item scale, the GAD-7, was used in this study [28]. Participants were asked how often they were bothered by each of the seven core symptoms of GAD with four response options: 0 = not at all, 1 = several days, 2 = more than half the days, and 3 = nearly every day, over the last 14 days. The response options were also calculated as continuous ordinal measure. Therefore, the range of scores of the GAD-7 was 0 to 21. The cut-off points for the categorization of the level of GAD symptoms were–‘0–4’ for minimal, ‘5–9’ for mild, ‘10–14’ for moderate, and ‘15–21’ for severe.

A standardized Bangla version of the GAD-7 was used for the study which was translated from the original English version. The standardization of the Bangla language was achieved by following ‘state of the art procedures’ for test translation [29].

*Sociodemographic*. Data on gender, age, grade in school, birth order, parent’s educational level, number of family members and residential setting were collected from the adolescents.

*Lifestyle*. Physical activity (PA), screen based sedentary behaviour (SBSB) and sleeping status were considered for determining the lifestyle of the adolescents. Three different PA levels were incorporated in the questionnaire: low-level PA (unintentional walking <30 min/d), moderate PA (walking or meditation/yoga ≥30 min/d) and vigorous PA (jogging, cycling, playing sports or gym workouts ≥60 min/d). Regarding SBSB, the adolescents were asked if they spent time on the social media like Facebook, Twitter, and Instagram, watched videos on YouTube or watched movies or sports. The pattern of their viewing videos/movies/sports and spending time on social media were further categorized in hours per day. High leisure screen time was described as >2 h/d, which conforms with the commonly used screen time guideline.

### Statistical analysis

Data were analysed using descriptive statistics as well as inferential statistics. To identify any significant relationships between the study variables, test statistics such as the χ^2^ test were used. Logistic regression models were used to detect any association of study variables with the outcome variables. In regression analysis, data were adjusted for various factors and were reported for the adjusted odds ratios (AORs) with 95% CIs. The level of significance was set at p<0.05. The Statistical Package for the Social Sciences software for Windows, version 22.0 (IBM, Armonk, NY, USA) was used to analyse all data.

### Ethics approval and consent to participate

Ethical approval was taken from Institutional Review Board, Jahangirnagar University (Ref No: BBEC, JU/ M/ 2019 (8) 3). Besides, all participants read, understood and signed a written consent form at the time of survey data collection.

## Results

### Sociodemographic characteristics of the study participants

**Table 1** demonstrates the sociodemographic characteristics of the study participants. More than half (51.6%) of the study participants were female and a third (32.2%) of the study participants were aged 14 years. Nearly half (43.3%) of the fathers of the respondents were service-holders while most (81.5%) of the mothers of the respondents were homemakers. Most (62.0%) of the participants came from small families (≤ 4 members).

**Table 1:** Sociodemographic characteristics of the participants.

Variables		Frequency	Percentage
**Gender**			
	Male	1120	48.4
	Female	1193	51.6
**Age in years**			
	12	85	3.7
	13	362	15.7
	14	745	32.2
	15	703	30.4
	16	358	15.5
	17	60	2.6
**Student grade**			
	Class 8	825	35.7
	Class 9	983	42.5
	Class 10	505	21.8
**Birth order**			
	1^st^	1183	51.1
	2^nd^	775	33.5
	≥3rd	355	15.3
**Father’s level of education (n = 1450)**			
	Primary education	177	7.7
	Secondary/ Higher secondary education	490	21.2
	Graduation/above	783	33.9
**Father’s occupation**			
	Business	793	34.3
	Service holder	1002	43.3
	Doctor/ Engineer/ Teacher/ Advocate/ Banker	260	11.2
	Other services	182	7.9
	No job/ Not applicable	76	3.3
**Mother’s level of education (n = 1486)**			
	Primary education	257	11.1
	Secondary/ Higher secondary education	680	29.4
	Graduation/above	549	23.7
**Mother’s occupation**			
	Homemaker	1884	81.5
	Doctor/ Engineer/ Teacher/ Advocate/ Banker	146	6.3
	Other services	246	10.6
	No job/ Not applicable	37	1.6
**Total no of family members**			
	≤ 4 members	1433	62.0
	≥ 5 members	880	38.0
**Residence**			
	Urban	830	35.9
	Semi-urban	743	32.1
	Rural	740	32.0

#### Prevalence and severity of anxiety

**Fig 1:**
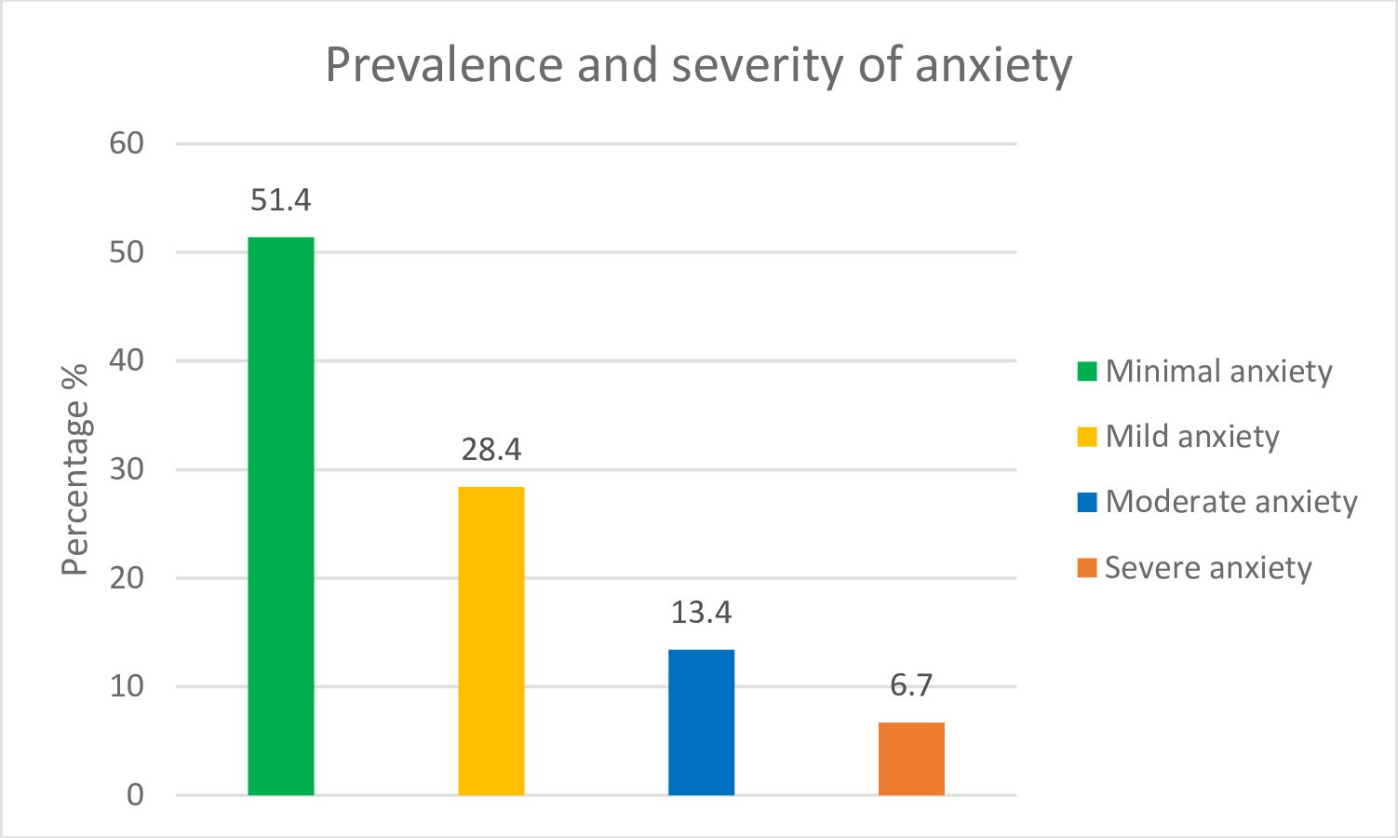
Prevalence and severity of anxiety among school adolescents in Dhaka, Bangladesh.

The prevalence and severity of anxiety among the participants are shown in **Fig 1**. It was found that 6.7% of the participants suffered from severe anxiety, while 13.4% were found to be experiencing moderate anxiety. 28.4% of the participants were found to experience mild anxiety, and 51.4% suffered from minimal anxiety at the time of data collection.

#### Association of sociodemographic variables with adolescent anxiety

The association between sociodemographic variables and anxiety has been illustrated in **Table 2**. This table presents that a significantly (χ2 = 16.07; p<0.001) higher proportion of female students (49.9%) were suffering from anxiety than their male counterparts (40.1%). Besides, anxiety was found to be more prevalent among students of 15 years (33.3%), followed by the students aged 14 years (29.0%). Age was found to be statistically highly significant (χ2 = 22.05; p = 0.001) with anxiety. Again, students of grade 9 (45.3%) were found to be suffering more from anxiety than those of grades 8 and 10. Chi-square analysis shows that a significant (χ2 = 8.40; p = 0.015) relationship exists between anxiety and students’ grade. In terms of the participants’ residential setting, 47.9% of the urban students, 33.9% of the semi-urban students and 18.2% of the rural students were found to be suffering from anxiety, and the residential setting was found to have a statistically significant (χ2 = 58.38; p<0.001) association with anxiety. In addition, anxiety was significantly (χ2 = 5.37; p = 0.020) linked to the number of family members. 57.3% of the students from families with ≤4 members were found to be suffering from anxiety. Again, the fathers’ occupation was found significantly (χ2 = 7.90; p = 0.019) associated with students’ anxiety. 58.5% of the students whose fathers completed graduation or above were found to be suffering from anxiety, and 27.1% of the students whose fathers completed the secondary or higher secondary level of education were suffering from anxiety. However, no significant association was found between the mothers’ educational level and anxiety.

**Table 2:** Association of sociodemographic variables with students’ anxiety.

Variables		Anxiety^a^	
		Frequency (% within column)	χ^2^ value (p-value)
**Gender**			
	Male	187 (40.1)	16.07 (<0.001)**
	Female	279 (49.9)	
**Age in years**			
	12	10 (2.1)	22.05 (0.001)**
	13	57 (12.2)	
	14	135 (29.0)	
	15	155 (33.3)	
	16	90 (19.3)	
	17	19 (4.1)	
**Student grade**			
	Class 8	140 (30.0)	8.40 (0.015)*
	Class 9	211 (45.3)	
	Class 10	115 (24.7)	
**Birth order**			
	1^st^	236 (50.6)	0.63 (0.729)
	2^nd^	153 (32.8)	
	≥ 3^rd^	77 (16.5)	
**Father’s level of education (n = 1450)**			
	Primary education	43 (14.4)	7.90 (0.019)*
	Secondary/ Higher secondary education	81 (27.1)	
	Graduation/above	175 (58.5)	
**Mother’s level of education (n = 1486)**			
	Primary education	49 (16.4)	0.48 (0.786)
	Secondary/ Higher secondary education	134 (45.0)	
	Graduation/above	115 (38.6)	
**Total no of family members**			
	≤ 4 members	267 (57.3)	5.37 (0.020)*
	≥ 5 members	199 (42.7)	
**Residence**			
	Urban	223 (47.9)	58.38 (<0.001)**
	Semi-urban	158 (33.9)	
	Rural	85 (18.2)	

^a^The cut-off of GAD-07 ≥10 is used for analysis:

** p value is significant at p < 0.01

* p value is significant at p = 0.05

#### Association of lifestyle variables with adolescent anxiety

**Table 3** demonstrates the association between students’ anxiety and lifestyle variables. Findings from this table revealed a statistically significant (χ2 = 7.00; p = 0.008) relationship between anxiety and involvement in PA. 23.1% of the students who were not involved in PA were found to be suffering from anxiety. Besides, 22.7% of them involved in irregular PA were found to experience anxiety. A statistically significant (χ2 = 6.06; p = 0.014) relationship was found between the regularity of PA and anxiety. In terms of the duration of daily PA, 21.3% of the participants who reported to do PA <30 minutes per day were suffering from anxiety. Again, 25.3% of the students who reported to do their PA in the evening were found to be suffering from anxiety. Use of social media had a high statistically significant (χ2 = 14.65; p<0.001) relationship with anxiety. Of the students who reported using social media, 22.7% were suffering from anxiety. Of the students with SBSB of >2 hours per day, 25.2% were found to be suffering from anxiety. This variable was found to have a high statistically significant (χ2 = 27.54; p<0.001) association with anxiety. In addition, 37.1% students who were dissatisfied with their daily sleep were found to be suffering from anxiety, and a statistically significant (χ2 = 212.40; p<0.001) relationship was found between sleep satisfaction and anxiety. Sleep duration had a prominent significant (χ2 = 43.14; p<0.001) association with anxiety. 26.8% and 19.6% of students who had respectively short (<7 hours/day) and long (>9 hours/day) sleep duration were found to be suffering from anxiety.

**Table 3:** Association of lifestyle variables with adolescent’s depression.

Variables		Anxiety^a^	
		Frequency (% within variable)	χ^2^ value (p- value)
**Physical activity (PA)**			
**Involved in PA**			
	Yes	272 (18.5)	7.00 (0.008)**
	No	194 (23.1)	
**Regular PA**			
	Yes	264 (18.5)	6.06 (0.014)*
	No	202 (22.7)	
**Duration of daily PA (n = 1472)**			
	<30 min/day	308 (21.3)	3.51 (0.173)
	30–60 min/day	94 (17.8)	
	>60 min/day	64 (18.7)	
**PA time (n = 1472)**			
	Early morning of the day	87 (18.4)	11.39 (0.010)*
	Late afternoon of the day	137 (17.3)	
	Evening of the day	40 (25.3)	
**Screen based sedentary behaviour (SBSB)**			
**Use of social media (e.g., Facebook)**			
	Yes	323 (22.7)	14.65 (<0.001)**
	No	143 (16.1)	
**Screen based recreation (Movie, video game etc.)**			
	Yes	420 (19.8)	2.01 (0.157)
	No	46 (24.1)	
**Duration of daily SBSB**			
	≤2 hours/day	215 (16.3)	27.54 (<0.001)**
	>2 hours/day	251 (25.2)	
**Sleep quality**			
**Satisfaction about daily sleep**			
	Yes	174 (11.4)	212.40 (<0.001)**
	No	292 (37.1)	
**Sleep habit**			
	Short sleep duration (<7 hours/day)	244 (26.8)	43.14 (<0.001)**
	Ideal sleep duration (7–9 hours/day)	194 (15.4)	
	Long sleep duration (>9 hours/day)	28 (19.6)	

^a^The cut-off of GAD 07 ≥10 is used for analysis

** p value is significant at p < 0.01

* p value is significant at p = 0.05

#### Association between predictive study variables and anxiety among school adolescents in Dhaka, Bangladesh

**Table 4** illustrates the logistic regression association between predictive study variables and anxiety. In this table, both bivariate analysis and multivariate analysis were presented. Adjusted estimate-1 was adjusted for all presented socio-demographic variables (age, gender, grade, residence) and adjusted estimate-2 was adjusted for level of PA, duration of daily screen time, satisfaction about daily sleep, sleep habit, and perceived weight category. After adjusting for sociodemographic and selected lifestyle variables, the odds ratios for variables changed slightly.

**Table 4:** Association between predictive study variables and anxiety among school adolescents in Dhaka, Bangladesh^a^.

Variables		Unadjusted estimates			Adjusted estimates ^b^			Adjusted estimates ^c^		
		Odds ratio	95% CI	p-value	Odds ratio	95% CI	p-value	Odds ratio	95% CI	p-value
**Socio-demographic variables**										
**Gender**										
	Female	1.52	1.24–1.87	<0.001	1.70	1.37–2.10	<0.001	1.44	1.15–1.82	0.002
	Male	1.00			1.00			1.00		
**Student grade**										
	Class 10	1.44	1.09–1.90	0.009	1.15	0.79–1.66	0.469	1.22	0.91–1.64	0.193
	Class 09	1.34	1.06–1.70	0.016	1.26	0.95–1.66	0.108	1.33	1.03–1.71	0.030
	Class 08	1.00			1.00			1.00		
**Age**										
	≥15 years	1.51	1.23–1.85	<0.001	1.39	1.06–1.84	0.020	1.31	1.05–1.64	0.016
	<15 years	1.00			1.00			1.00		
**Residence**										
	Urban	2.83	2.15–3.72	<0.001	2.96	2.24–3.91	<0.001	1.74	1.29–2.35	<0.000
	Semi-urban	2.08	1.56–2.77	<0.001	2.01	1.50–2.69	<0.001	1.47	1.08–1.99	0.014
	Rural	1.00			1.00			1.00		
**Lifestyle variables**										
**Involved in PA**										
	No	1.32	1.08–1.63	0.008	1.23	1.03–1.60	0.025	1.49	0.67–3.31	0.332
	Yes	1.00			1.00			1.00		
**Regular PA**										
	No	1.30	1.05–1.59	0.014	1.27	1.02–1.58	0.035	1.31	1.05–1.63	0.018
	Yes	1.00			1.00			1.00		
**Level of PA**										
	Inactive/ Low PA	1.22	0.99–1.51	0.065	1.04	0.83–1.32	0.727	0.90	0.67–1.21	0.490
	Moderate to vigorous PA	1.00			1.00			1.00		
**Use of social media (e.g., Facebook)**										
	Yes	1.53	1.23–1.90	<0.001	1.46	1.16–1.84	0.001	1.25	0.96–1.64	0.102
	No	1.00			1.00			1.00		
**Duration of daily screen time**										
	>2 hours/day	1.72	1.40–2.11	<0.001	1.54	1.24–1.91	<0.001	1.51	1.21–1.88	<0.001
	≤2 hours/day	1.00			1.00			1.00		
**Satisfaction about daily sleep**										
	No	4.57	3.69–5.66	<0.001	3.91	3.13–4.89	<0.001	3.79	3.02–4.76	<0.001
	Yes	1.00			1.00			1.00		
**Sleep habit**										
	Short sleep duration	2.02	1.63–2.49	<0.001	1.82	1.47–2.27	<0.001	1.34	1.06–1.69	0.014
	(<7 hours/day)									
	Long sleep duration	1.34	0.86–2.08	0.194	1.32	0.85–2.07	0.221	1.05	0.65–1.69	0.837
	(>9 hours/day)									
	Ideal sleep duration	1.00			1.00			1.00		
	(7–9 hours/day)									
**Body image dissatisfaction** ^**d**^										
	Yes	2.32	1.88–2.86	<0.001	2.05	1.65–2.54	<0.001	NA		
	No	1.00			1.00					
**Perceived weight category**										
	Over-weight/obese	2.15	1.69–2.75	<0.001	1.80	1.40–2.32	<0.001	1.75	1.35–2.27	<0.001
	Underweight	2.93	2.15–3.99	<0.001	2.82	2.05–3.88	<0.001	2.37	1.70–3.28	<0.001
	Normal	1.00			1.00			1.00		

^a^Estimates are based on binary logistic regression with anxiety, GAD 07≥10 scores, as dependent variable.

^b^ Adjusted for all presented socio-demographic variables (age, gender, grade, residence) in the table.

^c^ Adjusted for regular PA, duration of daily screen time, satisfaction about daily sleep, sleep habit, and perceived weight category.

^d^ Body image dissatisfaction was highly correlated with perceived weight category (r = -0.88) and removed from the multivariate model.

*NA* Not applicable (excluded from the multivariate model since their P value was greater than 0.1

In adjusted estimate-1, it was found that female adolescents were 1.70 times (95% CI: 1.37–2.10) more at risk of suffering from anxiety than their male counterparts. Again, adolescents of higher age (≥15 years) had higher risks (AOR: 1.39; 95% CI: 1.06–1.84) of suffering from anxiety than younger adolescents. Urban adolescents were found to be 2.96 times (95% CI: 2.24–3.91) more at risk of suffering from anxiety than the rural adolescents, while adolescents of the semi-urban area had 2.01 times (95% CI: 1.50–2.69) greater risks of experiencing anxiety than their rural counterparts.

In adjusted estimate-2, it has been found that adolescents involved in irregular PA had 1.31 times (95% CI: 1.05–1.63) higher risks of suffering from anxiety than those involved in regular PA. Again, adolescents with a higher screen time (>2 hours/day) had 1.51 times (95% CI: 1.21–1.88) greater risks of suffering from anxiety than those with a lower screen time. Besides, adolescents who were dissatisfied with their daily sleep were 3.79 times (95% CI: 3.02–4.76) more at risk of suffering from anxiety than those satisfied with their daily sleep. Adolescents who perceived their body weight as underweight were more at risk (AOR: 2.37; 95% CI: 1.70–3.28) of suffering from anxiety than those who perceived their body weight as normal.

## Discussion

This study indicates that mild to severe anxiety is common among school adolescents in Bangladesh. Almost half (48.5%) of the participants were found to be suffering from anxiety. This prevalence is much higher than that (2.2%) reported in another study conducted upon 315 adolescents in the Dhaka city in Bangladesh in 2020 [30]. The prevalence of anxiety among adolescents in the current study is much higher than that (16.4%) reported in yet another study conducted in 2020 upon 622 adolescents in Bangladesh [31]. However, the prevalence of anxiety found in the present study is lower than that reported in a 2019 study conducted upon 590 university students (58.1%) in Bangladesh [32]. The difference in the prevalence might be explained in that the undergraduate university students who have just stepped into practical more challenging world encounter more practical problems than other adolescents. In the neighboring countries like India, the age-adjusted prevalence of anxiety among rural adolescents was 16.6%, which is much lower than that of the current study [33]. In another study, conducted upon school adolescents in Delhi, the prevalence of anxiety was 65.3%, which is much higher than that of the present study [34]. The difference in the prevalence of anxiety reported in these studies might be attributed to the demographic setting, since the first one was conducted upon rural adolescents, while the later upon urban adolescents. In a 2017study conducted upon 1124 adolescents in Pakistan, the prevalence of anxiety was found to be 21.4%, which is much lower than that of the current study [14]. Again, the prevalence of anxiety in our study is much higher than that of another study conducted among 1556 participants in 2015 in the Malaysian context, where the prevalence of anxiety was only 8.2% [35]. Moreover, the prevalence of anxiety in our study is much higher than a study conducted upon 29,709 children and adolescents in Iran, where the lifetime prevalence of anxiety was 2.6% [36].

Studies have recorded variations in the prevalence of anxiety among adolescents in other regions of the world. For example, in a study on 1514 early adolescents in Spain, the prevalence of anxiety was 11.8%, which is quite lower than the that of the current study [37]. A 2019 study in the USA reported that among children and adolescents aged 3–17 years, the prevalence of anxiety was 7.1%, which is again much lower than that of the present study [9]. These differences in findings might be caused by differences in cultures across the world, sociodemographic factors, academic factors, familial factors, and so on. The high prevalence of anxiety among adolescents in this study could be attributed to sociodemographic and lifestyle factors. Also, the absence of standard assessment tools and inconsistent use of validated tools might have caused the differences in the prevalence of anxiety in similar settings like India and Bangladesh.

In terms of sociodemographic reasons, being female is the most significant reason as 60.8% of the female respondents (OR: 1.52) were found to be suffering from anxiety. There might be several reasons behind this, for example, going through a transitional period, being forced for marriage, academic pressure, living condition, financial condition of the family, and such-like. This high prevalence of anxiety among females is in keeping with the findings of some other studies [33, 38, 39]. This study also demonstrates that students of class 9 suffer most (45.3%) from anxiety, which is similar to the findings of other studies [40, 41]. Being students of secondary classes, poor or medium academic achievement, high academic pressure as well as the fear of upcoming board examinations might be the contributing factors in this regard. The regression analysis shows that students of mid to late adolescence (≥15 years) had higher odds (OR: 1.51) of suffering from anxiety than those of early adolescence, which might be caused by excessive academic pressure, social pressure, tension of going into adulthood, and many more. This study also shows that adolescents from the urban residential settings are the worst sufferers of anxiety (47.9%) since they were 2.83 times more likely to be suffering from anxiety compared to rural adolescents, and this finding is coherent with the findings of some other studies [42–44]. There are different reasons for higher prevalence of anxiety among urban adolescents, for example, urban household setting, low or no space for PA, parents’ indifference, high frequency of SBSB, very few or no siblings, and less family integration, among others.

In terms of lifestyle-related factors, the regression analysis demonstrates that not being involved in PA (OR: 1.32) or being involved in irregular PA (OR: 1.30) and doing low PA (OR: 1.22) significantly impact adolescent anxiety, and findings from other studies are also consistent with this finding [45, 46]. Studies have demonstrated positive outcomes of PA in relation to anxiety; when adolescents are involved in PA at moderate to high levels, it nullifies anxiety symptoms and disorders. Moreover, different physiological mechanisms, for example, neuroendocrine, anti-inflammatory, and antioxidant effects of PA, outsmart anxiety among adolescents. Besides, PA has different social, behavioral and psychological mechanisms which lessen the anxiety symptoms [47].

This study has found a significant association between social media use and anxiety among adolescents. As the regression analysis reveals, students who used social media had 1.53 times higher risks of developing anxiety than those who were not using it. Moreover, students whose daily screen time exceeds 2 hours per day were 1.72 times more likely to be suffering from anxiety than those whose screen time was less than that. Keles et al (2019) in their study stated that though there is no direct relationship between social media use and mental health problems, it impinges on the sleep quality and thus triggers mental health problems [48]. This is supported by the findings of the current study where it was found that adolescents who were not satisfied with their daily sleep were 4.57 times more likely to be suffering from anxiety than those with daily sleep satisfaction. Again, multitasking is common on social media, and studies found that online multitasking could lead to potential mental health difficulties. Another issue of concern is that the number of social media accounts is highly correlated with increasing levels of anxiety [48]. They found that adolescents who reported short sleep duration (<7 hours/day) were more than 2 times more likely to be suffering from anxiety than those who had ideal sleep duration (7–9 hours/day). Evidence suggests that adolescents with disturbed or short sleep duration undergo anxiety along with other mental and behavioral problems. There is evidence from experimental studies that short sleep duration reduces self-ratings of positive affect, and increases negative affect and negative mood in response to a challenge [49]. Findings of the current study suggest that adolescents who were dissatisfied with their body image were suffering more (OR: 2.32) from anxiety than their counterparts who perceived their body weight as obese/overweight (OR: 2.15) and underweight (OR: 2.93). Vannucci et al. in their study show that body image dissatisfaction leads to perceived pressure from society in order to adhere to sociocultural body ideals, which makes individuals consider these ideals to be their personal standard and self-worth. Such body ideal internalization gives rise to appearance-based social comparisons with others and body surveillance (i.e., thinking about how one’s body looks compared to others) [50]. Studies have showed that when adolescents underestimate or overestimate their weight, they engage in health risk behaviors which result in lower self-esteem and poorer body image. Different physiological factors that may play crucial roles in this case include rapid physical changes of puberty along with social pressures from the mass media or peers. These result in changes in their perception of body shape, body weight and appearance. The social preference for thinness presented in the media strongly influence in shaping adolescents’ body image, specifically the concept of ‘ideal body weight’, making it difficult for adolescents to accurately classify themselves appropriately in terms of weight [51].

## Strengths and limitations

Despite being one of the highly sensitive public health issues, adolescent anxiety has received surprisingly little research attention in the context of Bangladesh. Again, the previous studies on adolescent mental health mostly covered either urban or rural contexts, none of them being conducted in the urban, semi-urban and rural settings simultaneously. To the best of our knowledge, this is one of the few studies conducted upon urban, semi-urban and rural adolescents to investigate the prevalence of anxiety, one of the common mental health disorders, in Bangladesh. This study was conducted over a one-year period, following a pilot study conducted upon the urban and semi-urban adolescents of the study area [52]. Therefore, the findings of this study could be useful for further research initiatives in the area as well as policy formulation in this regard. Further, this research ensured the field implementation procedures in a very comprehensive and rigorous way.

Nevertheless, this study has some limitations too. In this cross-sectional study, data were collected at one point of time, and hence in some cases the data might not be representative enough. Besides, the data consist of self-reported information, which almost always involves the risk of recall bias. On top of that, the participants represented a small portion of the adolescent population in the district of Dhaka. Therefore, the findings of the current study might not be generalizable to other districts of Bangladesh. Therefore, there is need to conduct large-scale longitudinal studies on the same cohort which will cover the entire district of Dhaka to obtain findings generalizable to similar contexts in Bangladesh.

## Conclusion

The findings of this study indicate that anxiety is prevalent among adolescents in Bangladesh, and certain sociodemographic as well as lifestyle-related factors are playing crucial roles behind the high prevalence of anxiety among this tender population. More large-scale, representative, and longitudinal research studies are needed to explore the actual scenario of adolescent anxiety in the country.

## Supporting information

S1 Data Anxiety among adolescent final data sheet. (SAV)

S1 File Survey questionnaire. (PDF)
