# Supplementary material for: Anxiety among urban, semi-urban and rural school adolescents in Dhaka, Bangladesh: Investigating prevalence and associated factors
Source: PLoS One. 2022 Jan 21;17(1):e0262716. doi: 10.1371/journal.pone.0262716 (PMC8782381; doi:10.1371/journal.pone.0262716)
Supplement: S1 File — (PDF) [file pone.0262716.s002.pdf]

Investigating Prevalence of and Factors Associated with Anxiety Disorder among Urban, Semi-urban and Rural School Adolescents in Dhaka District of Bangladesh

Section A: Socio-Demographic Information

Serial no.	Questions	Answer/Coding categories
A_1	What is your class/grade?	
A_2	Mention your age in years	
A_3	What is your gender?	1=Male<br>2=Female<br>3=Others
A_4	What is your birth order?	1= First child<br>2= Second child<br>3= Third child<br>4= Others (Please specify)<br>--------------------------------
A_5	What is your father's occupation?	
A_6	What is father's educational qualification?	
A_7	What is your mother's occupation?	
A_8	What is your mother's educational qualification?	
A_9	Please mention the number of your family members	

Section B: Quality of Health (Self-reported health/body image)

Serial no.	Questions	Answer/coding categories
B_1	Are you satisfied with your current sleeping manner?	1= Yes<br>2= No
B_2	At present, how many hours do you sleep?	----------------- Hours
B_3	Do you have any dissatisfaction with your body weight?	1=Yes<br>2=No
B_4	Body image:<br>How you perceive your current body weight?	1= Underweight<br>2= Normal/Healthy Weight<br>3= Overweight<br>4= Obese

Section C: Physical Activity

C_1	Do you have practice to do any physical activity?	1=Yes<br>2=No
C_2	If yes, do you do PA regularly?	1=Yes,<br>2=No<br>3= Sometimes
C_3	Over the last 7 days, how often did you involved in PA? (mention days)	
C_4	On average, how long do you spend in PA in a day?	1= less than 30 min.<br>2= 30min to 1 hour.<br>3= 1 to 2 hours.<br>4= More than 2 hours
C_5	By which time of the day would you like to do PA?	1= Early morning of the day (between 5 to 8 am)<br>2= Late afternoon of the day (between 4 to 6 pm)<br>3= Evening of the day (between 6 to 9 pm)

Section D: Screen Based Sedentary Behavior

D_1	Which kind of cell phone are you using?	1= Android<br>2= iPhone<br>3= Normal Phone<br>4= Don't use any phone
D_2	Are you using an type of social media (like, Facebook, Twitter, Instagram etc.)?	1=Yes<br>2=No
D_3	On an average how long do you spend social media?	(a) Daily (h)
D_4	Do you watch movie/play/live or YouTube video game for recreation?	1=Yes<br>2=No
D_5	If yes, mention when and how long?	(a) Daily (h)

Section E: Psychological Health

Instruction: For the last 14 days, how much you have felt towards the following statements. Please put a tick mark (✓) in the respective columns.

Generalized Anxiety Disorder Assessment (GAD-7)					
Serial no.	Over the last 2 weeks, how often have you been bothered by any of the following problem?	Not at all (0)	Several days (1)	More than half of the days (2)	Nearly every day (3)

E_1	Feeling nervous, anxious				
E_2	Not being able to sleep or control worrying?				
E_3	Worrying too much about different things?				
E_4	Trouble relaxing?				
E_5	Being so restless that it is hart to sit still?				
E_6	Becoming easily annoyed or irritable?				
E_7	Feeling afraid as if something awful might happen?				
